# Diagnosis and management of asthma – Statement on the 2015 GINA Guidelines

**DOI:** 10.1007/s00508-016-1019-4

**Published:** 2016-07-01

**Authors:** Fritz Horak, Daniel Doberer, Ernst Eber, Elisabeth Horak, Wolfgang Pohl, Josef Riedler, Zsolt Szépfalusi, Felix Wantke, Angela Zacharasiewicz, Michael Studnicka

**Affiliations:** 1 Allergy Center Vienna West, Hütteldorfertraße 46, 1150 Vienna, Austria; 2 Wilhelminen Hospital, Department of Internal and Pulmonary Medicine, Teaching Hospital of the Medical University of Vienna, Montleartstraße 37, 1160 Vienna, Austria; 3 Clinical Department of Pediatric Pulmonology and Allergology University Clinic for Pediatric and Adolescent Medicine, Graz University, Auenbruggerplatz 34/2, 8036 Graz, Austria; 4 Pediatric Pulmonology/Allergology, Department of Pediatric and Adolescent Medicine Innsbruck, Anichstr. 35, 6020 Innsbruck, Austria; 5 Department of Respiratory and Lung Diseases, Hietzing Hospital, Karl Landsteiner Institute for Experimental and Clinical Pulmonology, Wolkersbergenstraße 1, 1130 Vienna, Austria; 6 Department of Pediatric and Adolescent Medicine, Kardinal Schwarzenberg Hospital, Kardinal-Schwarzenbergstraße 2–6, 5620 Schwarzach, Austria; 7 Clin. Department for Pediatric Pulmonology, Allergology and Endocrinology, University Clinic for Pediatric and Adolescent Medicine, Währinger Gürtel 18–20, 1090 Vienna, Austria; 8 Floridsdorf Allergy Center, Franz Jonas Platz 8/6, 1210 Vienna, Austria; 9 Department of Pediatric and Adolescent Medicine, Wilhelminen Hospital, Montleartstr.37, 1160 Vienna, Austria; 10 University Clinic for Pulmonology, Hospital Salzburg, University Hospital of Paracelsus Private Medical University, Müllner Hauptstraße 48, 5020 Salzburg, Austria

**Keywords:** asthma, guidelines, diagnosis, treatment, GINA

## Abstract

This statement was written by a group of pulmonologists and pediatric pulmonologists belonging to the corresponding professional associations ÖGP (Austrian Society for Pulmonology) and ÖGKJ (Austrian Society for pediatric and adolescent medicine) to provide a concise overview of the latest updates in the 2015 GINA Guidelines and to include aspects that are specific to Austria.

## Introduction

Asthma is one of the world’s most common chronic diseases and also represents an important cost factor for health-care systems. The Global Initiative for Asthma (GINA) has published updated guidelines for the diagnosis and therapy of asthma in children and adults annually for over 10 years [4]. This rather voluminous document (>100 pages) in English can be difficult to implement for practicing physicians and in part also has to be adapted to local conditions.

This statement was written by a group of pulmonologists and pediatric pulmonologists belonging to the corresponding professional associations ÖGP and ÖGKJ to provide a concise overview of the latest updates in the 2015 GINA Guidelines and to include aspects that are specific to Austria. For further details, please refer to the individual relevant sections of the GINA Guidelines, which are available online (http://www.ginasthma.org).

## Definition and diagnosis

### Definition

Asthma is a heterogeneous, multifactorial disease with variable and mostly reversible respiratory pathway obstruction based on a chronic bronchial inflammatory reaction. The symptoms (cough, rhonchus, wheezing, chest tightness, or shortness of breath) are variable and correlated with expiratory flow limitation. Although bronchial hyperresponsiveness (BHR) is often present, the current GINA Guidelines no longer include it as a necessary or sufficient criterion for diagnosis.

### Phenotypes

Owing to the heterogeneity of the disease, a number of different phenotypes can be described. Distinguishing between them can be particularly relevant to the therapy in severe cases:

- Allergic asthma
- Nonallergic asthma
- Pediatric asthma/recurrent obstructive bronchitis
- Late-onset asthma
- Asthma with fixed airflow obstruction
- Obesity asthma
- Occupational asthma
- Asthma in the elderly
- Severe asthma

Classifications by other professional associations (*ERS/ATS*, European Respiratory Society/American Thoracic Society) tend to focus more on a combination of clinical and pathophysiological aspects (e. g., eosinophilic/neutrophilic asthma, severe allergic asthma etc.).

### Initial diagnosis

#### Diagnostic criteria and supplementary examinations

Asthma may be suspected if the patient has a positive medical history of recurrent dry coughing, especially at night, rhonchus, wheezing, chest tightness, or shortness of breath.

Lung function testing can confirm the diagnosis if an airway obstruction is found reversible based on an FEV_1 _(Forced expiratory Volume in 1 second) increase of >12 % and >200 ml (in adults) after administering 200–400 µg salbutamol. If there is clinical suspicion, but the lung function is normal, further bronchial challenge testing (e. g., with methacholine or indirect tests such as running exertion or inhalation of hyperosmolar solutions) may be helpful, especially to determine bronchial hyperresponsiveness in adults. Furthermore, an FEV_1_ increase of +12 % and >200 ml (in adults) after 4 weeks of anti-inflammatory therapy is considered diagnostic confirmation.

If an allergic trigger is suspected, an allergy diagnosis consisting of medical history, skin prick test, and/or definition of the specific IgE (Immunoglobuline group E) should be performed. This should also include sensitizations that are not clinically relevant because they can provide prognostic information.

Measuring fractional exhaled nitric oxide (FeNO) has not yet become an established practice in general asthma management and is not recommended in the current guidelines for a general therapy decision [6]. Nevertheless, in the view of this statement’s authors, there are several indications in which an FeNO measurement makes sense, such as, for example: (1) as another component of asthma diagnosis in difficult cases (normal lung function, unclear symptoms), (2) to check on therapeutic adherence regarding inhaled corticosteroid (ICS), or (3) for the early detection of worsening asthma.

#### Differential diagnosis

The differential diagnosis for asthma in adults over 40 years of age should primarily consider chronic obstructive pulmonary disease (COPD chronic obstructive pulmonary disease), sinusitis, or gastroesophageal reflux disease (GERD). The distinction between asthma and COPD can be difficult since symptoms may overlap, change, or exist in parallel (ACOS Asthma-COPD overlap syndrome). In younger patients, acute infections, congenital malformations (respiratory or heart), or foreign body aspirations may be more prominent (Table 1).

**Table 1:** Common differential diagnoses for asthma

	≤5 years	6–11 years	12–39 years	40+ years
Recurrent viral infections	X			
Gastroesophageal reflux	X			
Congenital malformations (tracheomalacia, vascular ring etc.)	X			
Tuberculosis	X			
Protracted bacterial bronchitis	X			
Immunodeficiency	X			
PCD	X	X		
BPD	X	X		
Foreign body aspiration	X	X	X	
Congenital heart defects	X	X	X	
Cystic fibrosis	X	X	X	
Chronic cough (upper respiratory tract)		X	X	
Bronchiectasis		X	X	X
VCD			X	X
Hyperventilation			X	X
Alpha1-antitrypsin deficiency			X	X
COPD				X
Left ventricular heart failure				X
Drug-related cough				X
Parenchymatous lung disease				X
Pulmonary embolism				X
Central airway obstruction				X

According to GINA update 2015, modified by the author team

*PCD* primary ciliary dyskinesia, *BPD* bronchopulmonary dysplasia, *VCD* vocal cord dysfunction, *COPD* chronic obstructive pulmonary disease

## Follow-up

### Asthma follow-up: symptoms and minimization of future risks

Asthma therapy aims to achieve maximum freedom from symptoms (nocturnal awakening, need for rescue medication, activity restrictions) and, thereby, maximum quality of life (Table 2). This can in most cases be achieved with consistent therapy. Therapeutic adherence is a problem because patients often do not suffer in spite of poor lung function and discontinue their therapy without asking their physician once they have experienced a quick response. Regular lung function controls are necessary, and a questionnaire such as the Asthma Control Test (ACT) can provide additional help for evaluating therapeutic efficacy in relation to symptom control.

**Table 2:** Asthma control according to GINA for adults and children

Symptoms in the past 4 weeks	Asthma symptom control		
	Well-controlled	Partly controlled	Uncontrolled
Daytime symptoms more than 2×/week (or 1×/week^a^)	No criterion applies	1–2 criteria apply	3–4 criteria apply
Nocturnal awakening due to asthma (or coughing^a^) at any time			
Reliever >2×/week (or >1×/week^a^)			
Any limitation of daily activity due to asthma			

^a^In children ≤5 years

Risk minimization includes smoking cessation, optimal protection from contaminants in the workplace, and specific immunotherapy.

### Lung function control

A lung function test should be performed for diagnosis or prior to starting therapy. Follow-up should occur individually based on asthma severity/symptoms in intervals of 3–6 months. In addition to these recommendations of the GINA Guidelines, lung function testing, performed approximately 4–6 weeks after a change of therapy, has proven successful for controlling treatment efficacy. A minimally relevant change in the FEV_1_ is indicated as 10 %.

Spirometry can generally be performed in children from about 6 years of age. Prior to that, diagnosis and follow-up have to rely on clinical parameters (see “Preschoolers”). It is important to note that spirometry results tend to be normal in many children with asthma and only show FEV_1_ restrictions during exacerbations.

### Determination of asthma severity

The classification of asthma severity has fundamentally changed over the past years. Traditionally, the degree of severity (intermittent, mild, persistent etc.) was based on symptoms and the use of rescue inhalers and limited lung function. Strictly speaking, this classification only applied to untreated patients. Since the response to treatment is essential, GINA has recommended the concept of *asthma control* and *therapy steps* since 2006. Severity therefore is no longer a snapshot, but is established retrospectively after a treatment period of several months and can change over time. It results from the therapy step that is required to achieve asthma control (see Table 2):

- Mild asthma: therapy step 1 or 2
- Moderate asthma: therapy step 3
- Severe asthma: therapy step 4 or 5

**Fig. 1:**
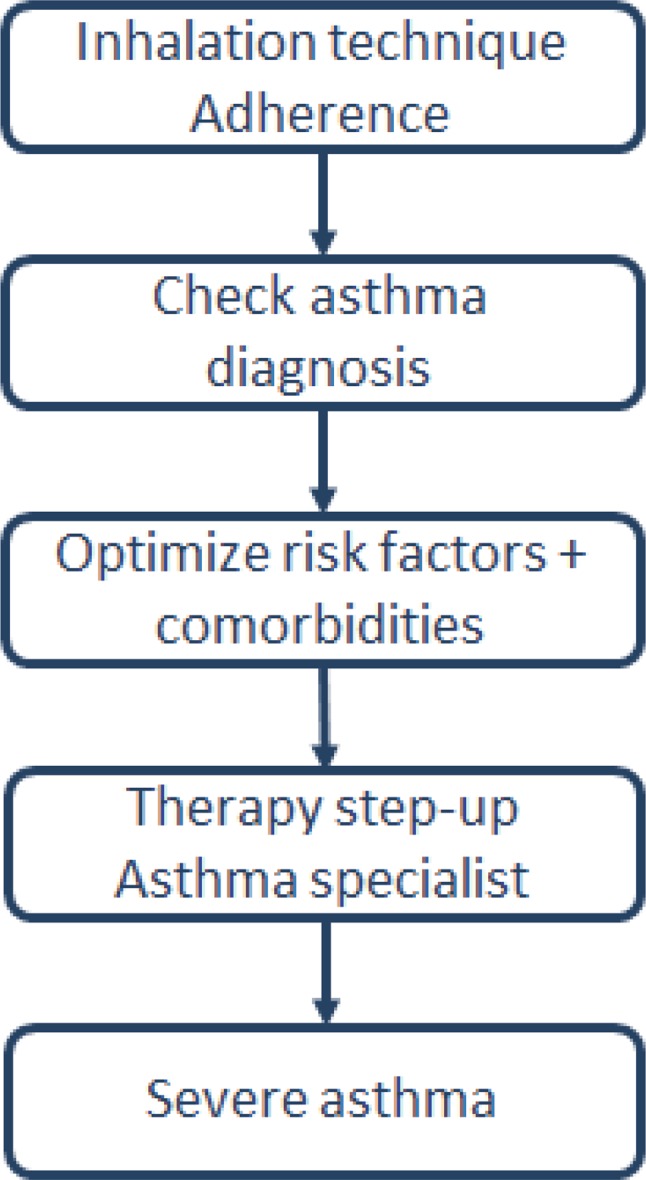
Investigation of a patient with poor symptom control or exacerbations despite therapy

In addition, the concept of severe asthma was clearly defined and differentiated from other terms such as uncontrolled or “difficult-to-treat” asthma. An exact definition can be found in a separate ERS/ATS guideline from 2014, to which GINA also makes reference [3]. “Severe asthma” is reserved for patients with actual therapy-resistant asthma after other factors such as poor inhalation technique and adherence to therapy, risk factors, continuous exposure to trigger factors (e. g., allergens, smoke) or co-morbidities have been excluded or optimally treated and the patient has received treatment from an asthma specialist for at least 3 months (see Fig. 1).

The World Health Organization term “untreated severe asthma” (common in countries with inadequate treatment options) also is no longer included in this definition of severe asthma.

It should also be noted that therapy steps 4 and 5 according to GINA or ERS/ATS have significant differences based on the defined limit values for high-dose ICS therapy (doses according to ERS/ATS are in part double compared with GINA). This has an indirect effect on the definition of severe asthma.

## Therapy and management

### Goals

General long-term objectives of asthma management include:

- Achieving symptom control and maintaining normal physical performance
- Minimizing the risk of exacerbations, fixed airway obstruction, and side effects of the therapy

Furthermore, individual goals should be discussed with the patient and taken into account. A good partnership between the patient and the physician is essential for effective asthma management. Physicians should have good communication skills to encourage patients to take part in decisions and to express their needs and concerns. Patient self-management and knowledge about asthma should be promoted with training, as studies have documented positive effects on morbidity in adults and children.

### Control-based asthma management

**Fig. 2:**
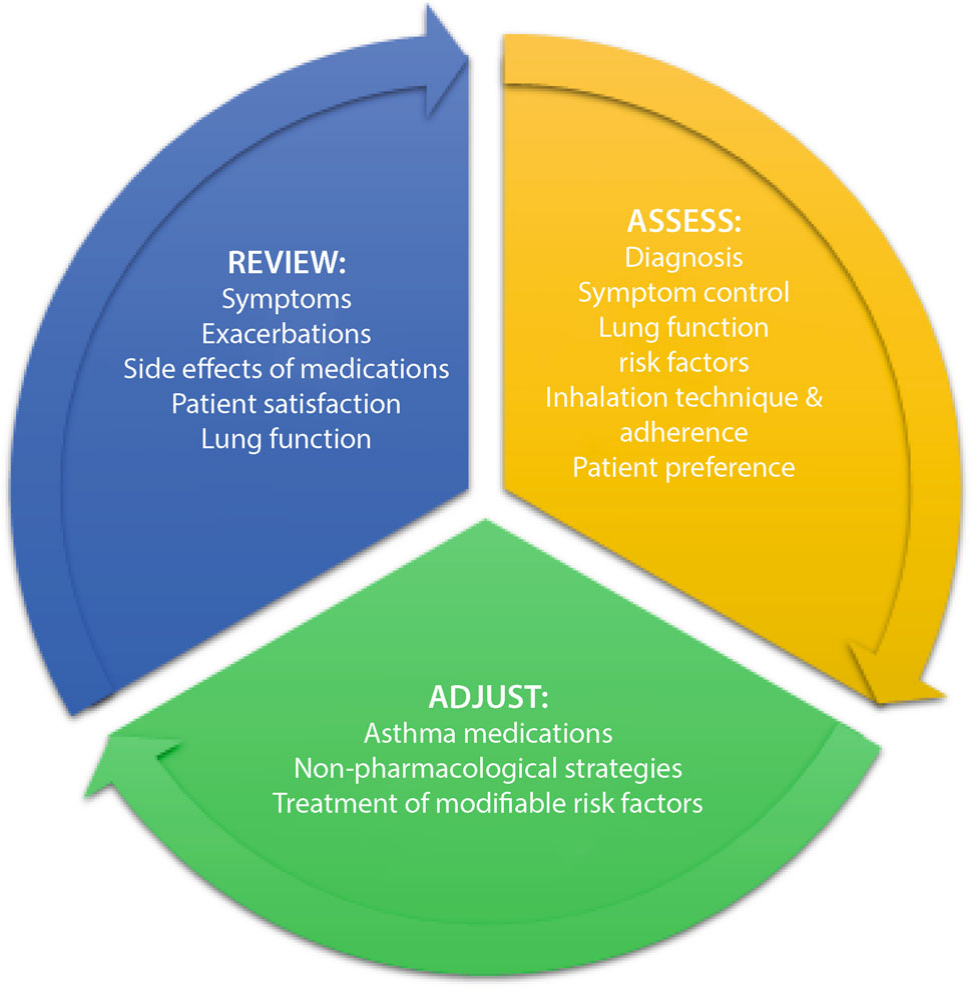
Cycle of asthma control

Modern asthma treatment (pharmacological and nonpharmacological therapy) is based on the concept of asthma control, which has been shown to improve the treatment success. This concept is based on a cycle of assess, adjust, and review (see Fig. 2).

Symptom control is usually associated with reduced asthma exacerbations. In the case of more severe forms, symptom control can occasionally not be paired with a reduced exacerbation rate. That makes it important to consider both factors of asthma control (symptoms and exacerbation risk).

As an alternative, other concepts such as therapy based on sputum or FeNO may be used in special cases, e. g., severe or difficult-to-treat asthma.

### Drug therapy

Inhalation therapy is the application of choice for asthma. It leads to higher local concentrations, fewer systemic side effects, and, accordingly, to generally very good tolerance compared with systemic application. Three pharmaceutical categories are generally distinguished for long-term treatment (Table 3):

- *Controller*: Taken regularly. Reduces inflammation and exacerbation risk and controls symptoms.
- *Reliever*: Taken as necessary to reduce symptoms in case of asthma exacerbations. Also used for the short-term prevention of exercise-induced bronchoconstriction. It is a key objective of asthma management to keep the need for reliever to a minimum.
- *Add-on*: Used in patients with severe asthma and persistent symptoms or exacerbations despite high-dose combination therapy with ICS and optimization of modifiable risk factors.

**Table 3:** Medication categories for asthma treatment

Controller	Reliever	Add-on therapy^a^
Inhaled corticosteroid (ICS) ICS/LABA (long-acting beta-2-agonist) combination Leukotriene receptor agonists (LTRA) Long-acting anticholinergics (LAMA) Methylxanthines (theophylline) Chromones (practically no longer in use)	Short-acting beta-2-agonists (SABA) Short-acting anticholinergics (LAMA)	Anti-IgE therapy Systemic/oral corticosteroids (OCS) Anti-IL5 therapy Special (phenotype-specific) treatments and interventions by specialized centers

^a^Add-on therapies should be prescribed by specialized physicians with experience in asthma management

### Initial treatment after diagnosis

To achieve the best possible results, a maintenance therapy with a controller should be initiated as quickly as possible after the diagnosis of asthma (improves lung function or reduces deterioration of lung function after an exacerbation; see Table 4). Since GINA 2014, a low-dose ICS therapy has been listed as an option for older children and adults at step 1 for specific symptom and risk constellations. For preschool children with intermittent symptoms, the initial therapy consists of an inhaled short-acting beta-2-agonist (SABA). Further therapeutic steps are based on the degree of asthma control.

**Table 4:** Controller therapy after initial diagnosis

Presentation	Recommended initial therapy^c^
– Symptoms or need for SABA <2×x/month – No nocturnal awakening – No risk factors^a^ for exacerbation – No exacerbation in the last 12 months	No controller
– Symptoms or need for SABA <2×/month – No nocturnal awakening – At least one risk factor^a^ for exacerbation or one exacerbation (with OCS therapy) in the last 12 months	Low-dose ICS
– Symptoms or need for SABA ≥2×/month – Nocturnal awakening ≥1×/month	Low-dose ICS
– Symptoms or need for SABA >2×/month	Low-dose ICS
– Symptoms on most days – Nocturnal awakening ≥1×/month – At least one risk factor^a^ for an exacerbation	Medium-/high-dose ICS or low-dose ICS/LABA^b^

^a^See Table 6

^b^ *Not* recommended as initial therapy for children up to age 11 years

^c^SABA used on demand in all cases

### Stepwise approach for adjusting asthma treatment

**Fig. 3:**
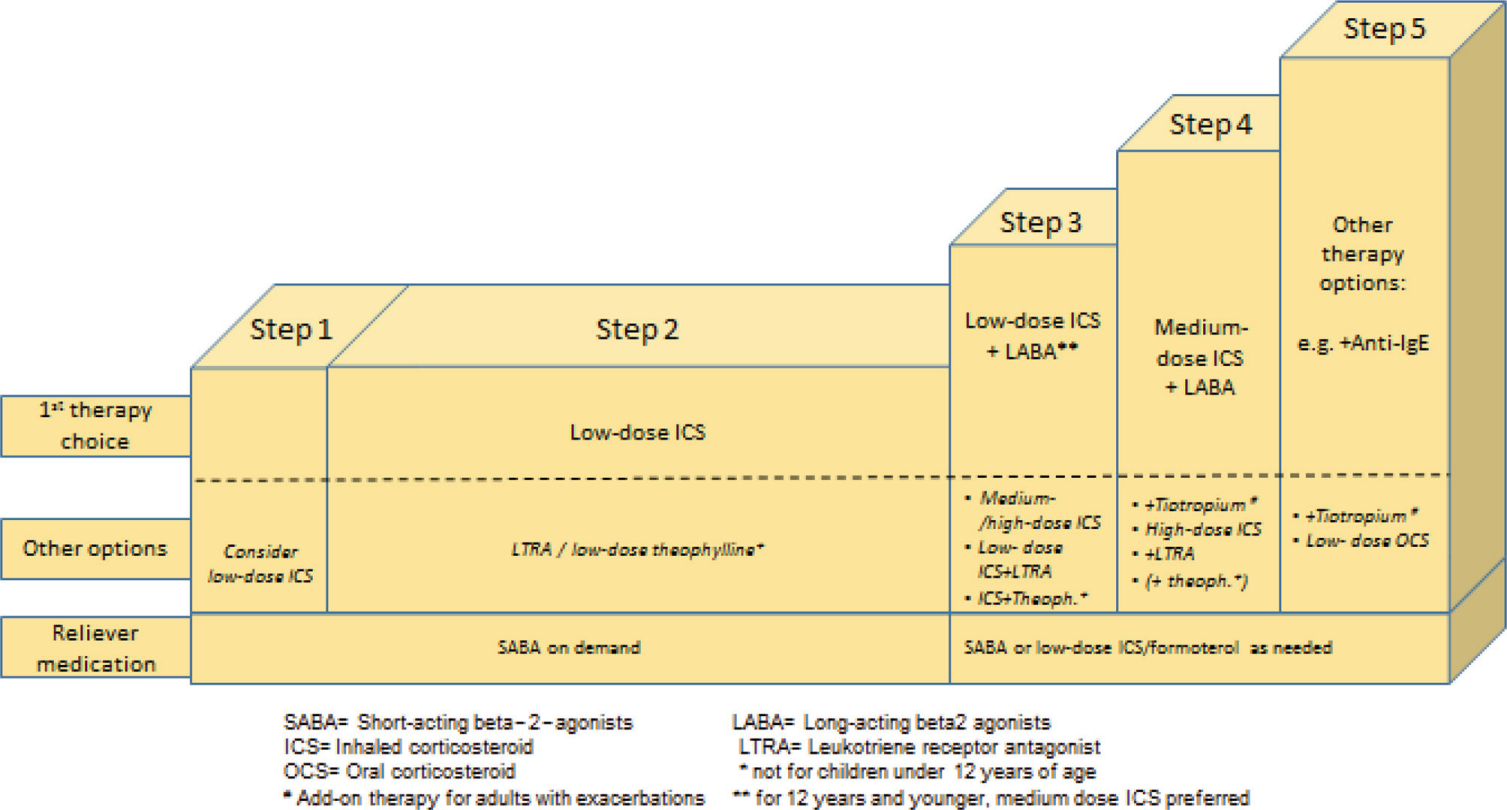
Step algorithm of asthma therapy for adults and children older than 5 years

The adjustment of asthma therapy is based on asthma control, and follows a step-up/step-down algorithm to increase or reduce the medication (Fig. 3). Regular follow-up should occur in a period of 2–3 months to optimize the treatment strategy. It is important to record symptom control, lung function, risk factors, inhalation technique, adherence, and nonpharmacological strategies on a regular basis.

The gold standard in asthma therapy is still a low-dose ICS as a controller together with an on-demand Short-acting beta-2-agonist (SABA). An LTRA (Leucotriene-receptor antagonist) can be tried as a second choice. There are also considerations that a combination product with low-dose ICS and long-acting beta-2-agonist (LABA) should be established in adults at this treatment stage (step 2) to ensure rapid treatment success. A further step-up from step 3 for adults calls for ICS–LABA combination therapy, with ICS in low doses. For children over the age of 12 years, an increased ICS dose is preferred over a combination therapy in this case. Two inhalers are also approved for additional rescue therapy: Formoterol/budesonide and formoterol/beclomethasone as basic therapy should be administered in the morning and at night and may also be inhaled by patients as needed (exacerbation).

The ICS concentration in the combination product increases with the severity of the disease. Tiotropium may now be added to the management regimen as an additional treatment option. The use of theophylline preparations is still defined in the guidelines, but they are rarely employed in practical situations. A further innovation in the step-up algorithm is the application of an add-on therapy (e. g., anti-IgE for patients with severe asthma, who show a corresponding allergic predisposition prior to a systemic steroid therapy.

In the same way as the step-algorithm provides for step-up options, it is important to reduce (step down) the therapy after the corresponding controls (after approximately 2–3 months) with good asthma control. Again, this requires a highly sensitive approach to quickly detect deteriorating symptoms and lung function, which may indicate an elevated risk of exacerbations. In principle, the goal should be to use the lowest ICS concentration that can guarantee optimal therapeutic success.

Asthma therapy for preschoolers basically has the same objectives as approaches for older children and adults and also follows a step scheme (step-up or step-down). In this age group, minimization of pharmaceutical side effects (e. g., body growth limitations from use of ICS) is especially important (see “Preschoolers”).

### Choice of inhaler

For children and adults, the choice of inhalers is based on individual skills (e. g., inspiratory flow) or barriers (e. g., arthritis, muscle weakness, impaired vision). Until early school age, most patients should use an inhaler with a suitable spacer. Oral/nasal masks should only be applied if the use of a mouthpiece is not possible (for reasons of age or compliance). In the age group of about 8 years and older, most patients use dry powder inhalers (DPI). If possible, patients should be involved in the choice of inhalers. Since there is no perfect tool, regular checks as well as training are necessary for the effective use of inhalers. The use of several different inhalers should be avoided to prevent confusion.

### Steroid dosage

The bulk of ICS effects can be achieved with low doses; few studies are available on dose–response relationships for conventional doses. Low daily doses are not associated with clinically relevant adverse effects, but high doses pose a larger risk of systemic side effects with prolonged application (although this risk is smaller than with systemic application). ICS should be titrated to find the minimum effective dose that achieves good symptom control and a minimal risk of exacerbation. The dose ranges for each age group are listed in Table 5.

**Table 5:** Daily dose equivalents of inhaled corticosteroids

Drug	Daily dose (mcg)		
	Low	Medium	High^a^
Adolescents and adults			
Beclomethasone dipropionate (CFC)	200–500	>500–1,000	>1,000
Beclomethasone dipropionate (CFC)	100–200	>200–400	>400
Budesonide (DPI)	200–400	>400–800	>800
Ciclesonide (HFA)	80–160	>160–320	>320
Fluticasone propionate (DPI)	100–250	>250–500	>500
Fluticasone propionate (HFA)	100–250	>250–500	>500
Mometasone furoate	110–220	>220–440	>440
Triamcinolone acetonide	400–1,000	>1,000–2,000	>2,000
School children			
Beclomethasone dipropionate (CFC)	100–200	>200–400	>400
Beclomethasone dipropionate (CFC)	50–100	>100–200	>200
Budesonide (DPI)	100–200	>200–400	>400
Budesonide (nebulizer)	250–500	>500–1,000	>1,000
Ciclesonide	80	>80–160	>160
Fluticasone propionate (DPI)	100–200	>200–400	>400
Fluticasone propionate (HFA)	100–200	>200–500	>500
Mometasone furoate	110	≥220–<440	≥440
Triamcinolone acetonide	400–800	>800–1,200	>1,200
Preschoolers			
Beclomethasone dipropionate (HFA)	100		
Budesonide (DA + spacer)	200		
Budesonide (nebulizer)	500		
Fluticasone propionate (HFA)	100		
Ciclesonide	160		

*CFC* chlorofluorocarbon as propellant, *MDI* metered-dose inhaler; *DPI* dry powder inhaler, *HFA* hydrofluoroalkane as propellant

^a^The guidelines of ATS/ERS on severe asthma use significantly higher cut-offs than specified here

### Specific immunotherapy

Although the GINA Guidelines are cautious in their recommendation of specific immunotherapy (SIT), an increasing volume of study data justifies its use in patients with mild-to-moderate, controlled allergic asthma [1]. It is important in each case that the allergen is a relevant asthma trigger (e. g., grass or birch pollen seasonally or house-dust mites as perennial allergens) and that the asthma is controlled (see Table 2). Particularly in the case of additional rhinoconjunctivitis, SIT can be taken into consideration for children from the age of 6 years with controlled asthma. Proper allergy diagnosis and the use of standardized vaccines are prerequisites for successful immunotherapy.

The treatment of asthma patients with specific immunotherapy should only be performed by doctors who are familiar with asthma management. Meticulous asthma control is required, especially in the step-up phase, for the problem-free performance of a specific immunotherapy.

**Table 6:** Risk factors for asthma exacerbations or asthma-related deaths

Increased risk of asthma exacerbations	Increased risk of death
Uncontrolled asthma symptoms	
High SABA use^a^	
	Use (currently or until recently) of OCS
Inadequate ICS use (not prescribed, wrongly inhaled, poor adherence)	No ICS inhalations; poor adherence to prescribed asthma medications, no asthma action plan (or not followed)
Low FEV_1_ (especially <60 % predicted)	
Serious psychosocial problems	
Exposed to: smoking, allergens (in case of existing allergy)	Existing food allergy
Sputum or blood eosinophilia	
Pregnancy	
Previously intubated or in an intensive care unit for asthma	Near-lethal episode with intubation and ventilation
≥1 serious exacerbations in the last 12 months	Hospitalization or presentation in the emergency room due to asthma in the last year

^a^Increased risk of death with >1 package of a salbutamol (or similar) MDI

Subcutaneous immunotherapy (SCIT) is still the gold standard for treatment. However, a significant reduction of steroid use and exacerbations was recently demonstrated with the use of a sublingual high-dose house-dust mite tablet in asthma patients [5].

### Nonpharmacological therapy

This section particularly refers to those patients who continue to suffer from asthma exacerbations in spite of presumably optimal or even maximum pharmacological therapy (see also “difficult-to-treat asthma”). However, many of the points listed, especially the avoidable factors (e. g., smoking, inactivity) are of relevance for all other asthma patients as well.

The identification of modifiable factors frequently leads to ultimate success. GINA discusses the factors to be considered and lists the corresponding leverage points. The detection of risk factors in the living environment of the patient and the implementation of improvement measures require a time-intensive doctor–patient dialog as well as the patient’s willingness to actively cooperate. Ultimately, successful asthma therapy is based on the complex interaction between doctor, patient, therapy, and the patient’s environment.

**Fig. 4:**
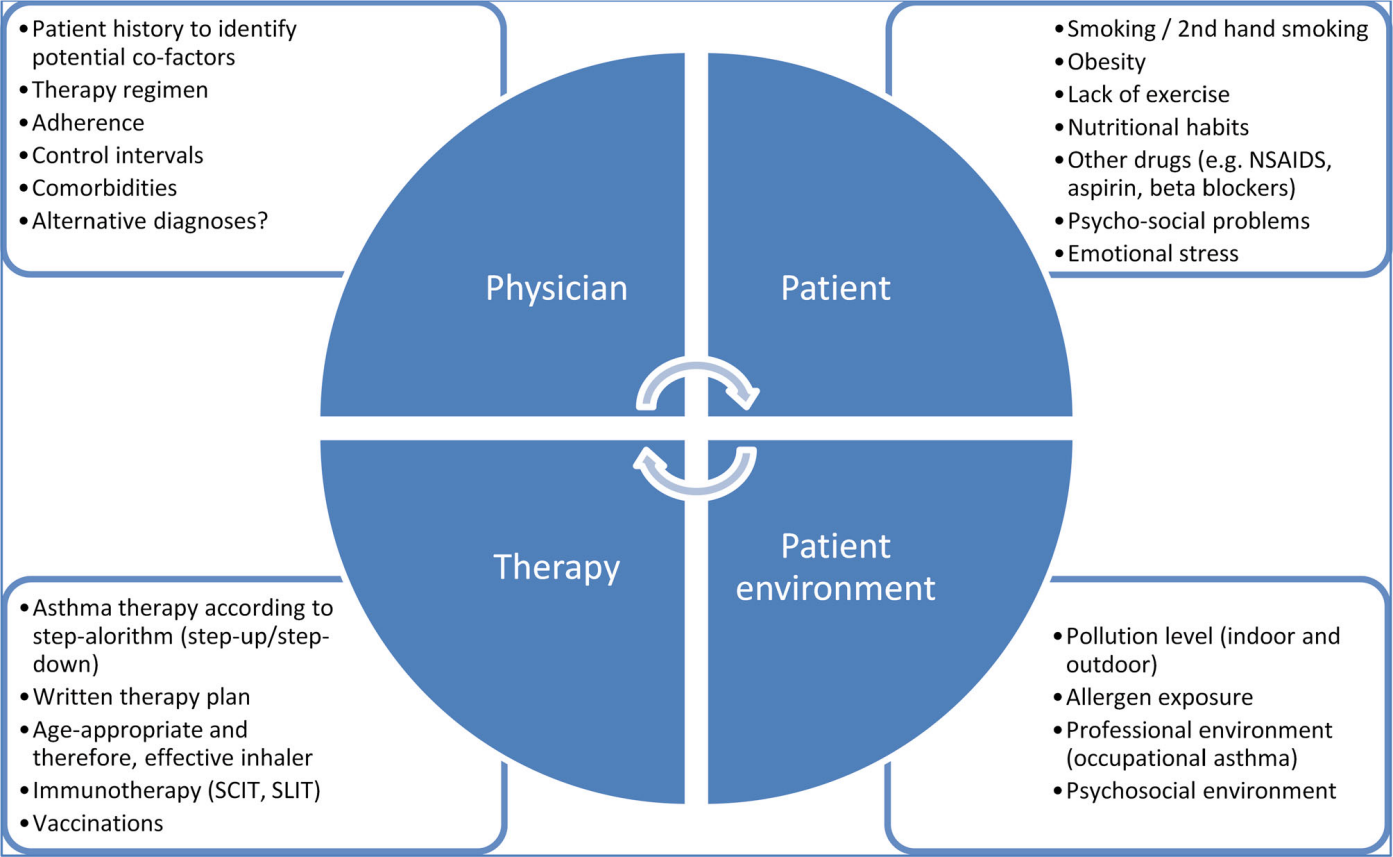
Uncontrolled asthma – additional therapeutic measures and modifiable factors

The main modifiable factors listed in GINA are briefly summarized in Fig. 4.

### Comorbidities

A number of health conditions can influence the course of asthma, thereby severely limiting asthma control and negatively affecting the patient’s life quality.

The presence of an inflammatory disease in the upper airways (*rhinitis*, rhinosinusitis) increases the risk of developing asthma. In all, 10–40% of patients with allergic rhinitis have asthma, suffer from more frequent exacerbations, and also need more rescue medication. Treating rhinopathy in patients with existing asthma is an important part of the therapy.

The heterogeneity of asthma can in part also be explained by the clinical characteristics of the comorbidities. This also includes *obesity* asthma, which generally represents a therapeutic challenge. Additional factors such as obstructive sleep apnea, GERD and small lung volumes complicate management of this special type of asthma, which has been intensively researched over the past few years. Common therapy concepts such as ICS are not successful, while a weight loss of 5–10% leads to a significant improvement of asthma control and life quality.

GERD is a trigger frequently found in adults, which is not only associated with increased cough episodes, but can also trigger asthma attacks. Asthma medications such as beta-2-agonists and theophylline preparations can aggravate these symptoms. In the case of uncontrolled asthma, it is generally recommended to look for GERD and to prescribe an empirical anti-reflux therapy for at least 6 weeks if GERD is suspected. In some cases, an endoscopic investigation or a 24-h pH measurement is required. The treatment consists of a high-dose PPI (protone-pump inhibitor) medication. Surgical procedures (fundoplication) in patients with therapy-resistant asthma must be considered as a final resort.

*Psychological disorders* are generally underestimated in asthma patients and often are associated with a low therapy adherence. Accordingly, these patients frequently experience exacerbations and have to seek emergency room treatment. Anxiety and depressive disorders should be professionally investigated, and patients should be trained to prepare for emergencies. Optimal pharmacological treatment is the basis for a better outcome in these patients.

In rare cases, *food allergies* are the triggers of asthma symptoms. Serious, even fatal reactions have been reported for patients with documented food-induced allergic reactions (anaphylaxis) and asthma. Because such patients frequently are allergic to peanuts or tree nuts, patients with food allergies should always be examined for the possible presence of asthma. Special allergological examinations can provide key information for the further management of these patients. In order to minimize the risk of an anaphylactic reaction, patients should receive a prescription for an adrenaline auto-injector and be instructed in its use.

### Disorders in specific populations

#### Adolescents

This section makes specific reference to the developmental changes occurring primarily in this age group (social, emotional, physical etc.). A range of different behavioral tendencies (such as smoking) must be taken into account. It is recommended to create an individual schedule that reflects the teen’s life situation and daily routine (topic: inhalation therapy – when and how often?).

#### Exercise-induced bronchoconstriction

Physical activities are an important trigger for the occurrence of asthma symptoms, although bronchoconstriction and the associated symptoms typically occur after exertion. Shortness of breath during exercise is more closely associated with a lack of physical fitness, obesity, or other illnesses. A well-founded pharmacological therapy can lead to significant improvements in exertion capacity, in which the administration of SABA can be sufficient to achieve substantial control. In some cases, regular treatment with ICS or ICS/LABA is required. Therapy with LTRA has also proven successful with this form of asthma. Targeted training measures and sufficient warm-up are important aspects of therapy.

#### Asthma in pregnancy

To avoid asthma exacerbations during pregnancy, patients should continue their treatment according to the GINA Guidelines. Therapeutic adherence can be problematic because pregnant women find it less acceptable to use ICS. If the asthma is well controlled during pregnancy, the risk of complications for mother or child is low. The use of ICS, SABA, and LTRA is not associated with an increased incidence rate of fetal abnormalities. The principle of avoiding asthma exacerbations with optimal therapy is particularly important during pregnancy. Continued ICS therapy stabilizes the asthma, while a discontinuation elevates the risk of clinical deterioration.

### Asthma training and self-management

GINA has a very clear position on the purpose of structured asthma training and nonpharmacological asthma management. The main content should be developed jointly and mainly concerns information on actions to take, training in self-management with recording of symptoms and possibly measurement of peak flow, the preparation of a written action plan, and regular follow-up for asthma control.

Training particularly focuses on the effective handling of the inhalation devices. Necessary steps include the selection of the most suitable inhaler for a specific age, reviewing inhalation technique, and demonstrations of correct application by the trainer. The patient should confirm the learning outcome by demonstrating the inhalation.

Therapeutic adherence should be checked at each visit, with recording of the possible causes of poor adherence. Around 50 % of all adults and children using long-term asthma therapy do not properly comply with their treatment regimen. How can adherence be verified? The guidelines make a few suggestions, which are generally based on “empathy” questions that are not moralizing or patronizing. The guidelines again emphasize the importance of shared decisions for an individually implementable therapy.

Information is part of every asthma training. It should be relevant for action and individually adapted to the development level of children. Training for preschoolers should be interactive, creative, and playful. It should be noted that information alone does not improve the outcome and should therefore only be part of a training that includes effective and practical aspects. It is recommended to address personal expectations, concerns, and fears so as to develop shared goals.

The communication of a self-management concept must reflect the personal situation of the family. Symptoms can be logged with a diary or an appropriate app. Given the low sensitivity and specificity of this method, peak flow measurement only makes sense in children who have severe or difficult-to-treat asthma.

The written asthma action plan that forms part of self-management should contain clear recommendations for the use of bronchodilation and anti-inflammatory therapy in acute and long-term management. In addition, it should be specified in detail how and when to seek medical assistance.

The benefits of telemedicine are seen as rather limited and should be primarily restricted to patients with severe forms of the disease. Regular medical consultation is regarded essential to implement the necessary aspects of pharmacological-based and nonpharmacological-based asthma management.

### Management of asthma exacerbations

An asthma exacerbation is an episode with increasing shortness of breath, cough, and wheezing involving restricted lung function. It is commonly caused by known triggers (respiratory infection, pollen, air pollution). This deterioration can be described in different terms, such as exacerbation, episode, acute severe asthma, or “flare-up.” In addition to the aforementioned trigger factors of an asthma exacerbation, they also – occasionally at the same time – represent key risk factors for asthma-related death (Table 6).

#### Diagnosis of exacerbations

An exacerbation is defined as a deterioration of lung function (FEV_1_) and/or the occurrence or deterioration of respiratory symptoms. While lung function constitutes an objective indicator, especially for determining the severity of the exacerbation, the frequency of respiratory symptoms is often a more sensitive parameter. Particular attention must be paid to patients who are less skilled at noticing or articulating their symptoms (children, male patients, patients with a near-lethal asthma event in the past).

The management of an asthma exacerbation occurs in stages and depends on the severity or the course. In principle, three stages can be distinguished:

- At home (self-management)
- At the family physician
- In the hospital/emergency room

#### Self-management (at home)

**Fig. 5:**
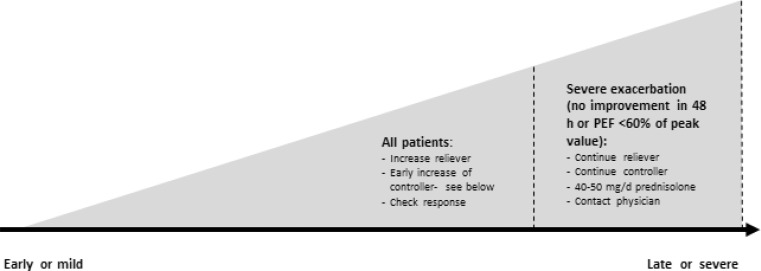
Self-management in case of exacerbations

All asthma patients should be trained in self-management and receive a written asthma action plan, which defines individual criteria for the modification of the controller therapy and typically pertains to symptoms and a drop in the PEF (Peak Expiratory Flow) (e. g., >20 % for more than 2 days). A general scheme is shown in Table 7 and Fig. 5.

**Table 7:** Modification of medication in the case of asthma exacerbation

Existing medication	Modification (for 1–2 weeks)
*Reliever (or C&R):* – SABA – Low-dose ICS/formoterol (C&R)	*Increase reliever:* – Increase SABA frequency – Increase reliever use (up to 72 µg/day formoterol)
*Controller:* – ICS/formoterol (C&R)^a^ – ICS + SABA – ICS/formoterol (C) + SABA – ICS/salmeterol + SABA	*Increase controller:* – Increase ICS/formoterol use (up to 72 µg/day formoterol) – Double ICS dose/increase to high-dose therapy step – Quadruple ICS/formoterol use (up to 72 µg/day formoterol) – Increase to high-dose ICS/salmeterol or ICS separately
*Any therapy*	*Add OCS and seek medical assistance:* – OCS for severe exacerbations (for criteria, see above) – Adults: 5–7 days 1 mg/kg/d prednisolone (max. 50 mg) – Children: 3–5 days 1–2 mg/kg/day prednisolone (max. 40 mg) – No wash-out phase required if administered <2 weeks – Double ICS dose/increase to high-dose therapy step – Quadruple ICS/formoterol use (up to 72 µg/day formoterol) – Increase to high-dose ICS/salmeterol or ICS separately

*C* as controller, *R* as reliever

^a^Not approved for children under 12 years of age

Patients should be examined by a physician within 1–2 weeks of an exacerbation. In general, the controller therapy can be reduced to the original step after 2–4 weeks.

#### Management by a general practitioner or pediatrician

In the medical setting, the following points need to be considered in the case of an exacerbation:

- Brief medical history and physical examination
- Assessment of exacerbation severity
- Prompt initiation of therapy

Medical history should include:

- Time and cause of exacerbation
- Severity of symptoms (physical performance and sleep)
- Signs of anaphylaxis
- Risk factors for asthma-related death (Table 6)
- Medication (reliever and controller, adherence, and recent changes)

**Fig. 6:**
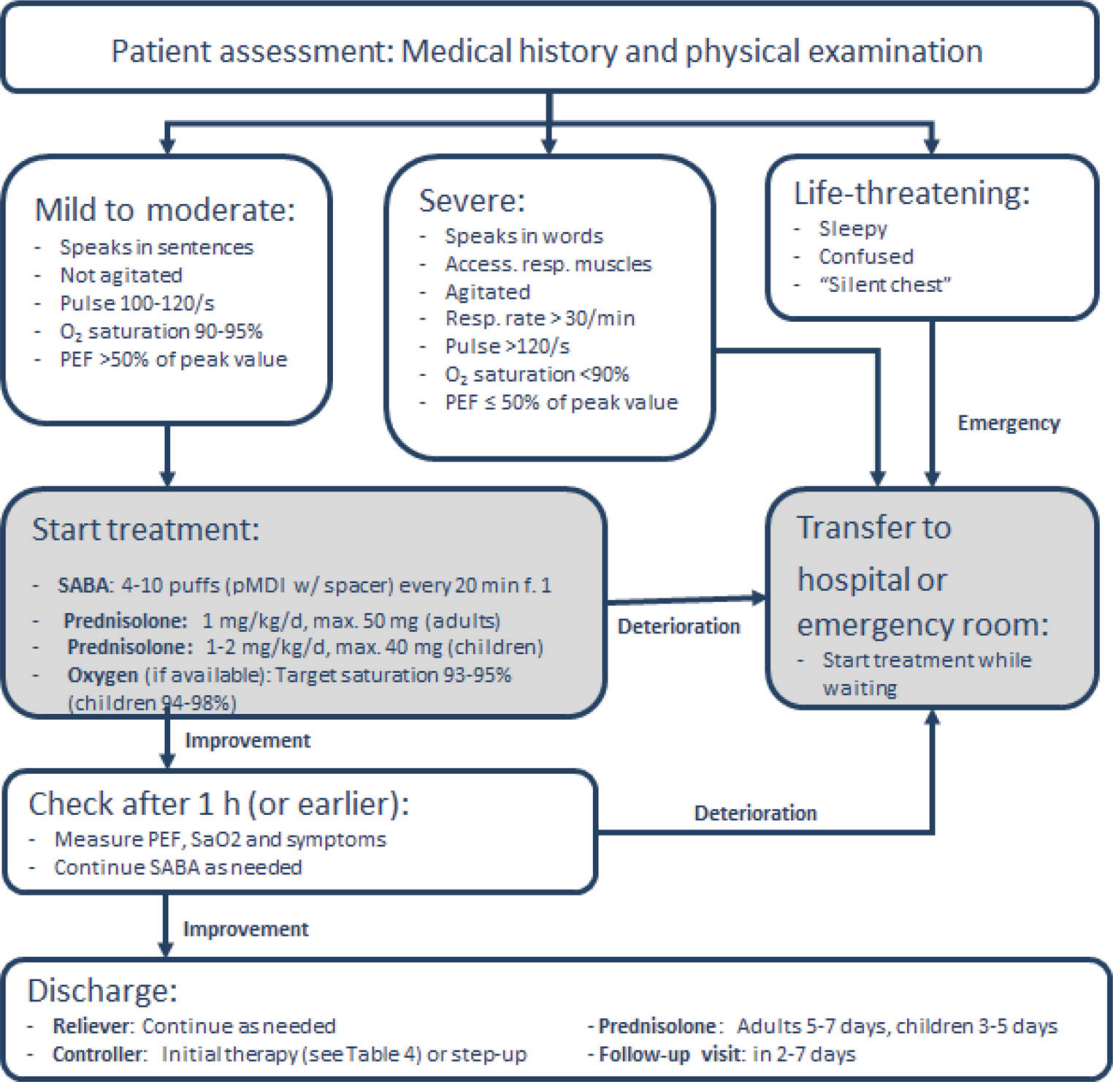
Severity signs of an asthma exacerbation

Clinical examination should include:

- Vital signs and signs of severity (see Fig. 6)
- Signs of complications (e. g., anaphylaxis, pneumonia, pneumothorax)
- Signs of alternative diagnoses (e. g., foreign body, pulmonary embolism, heart failure, dysfunction of the upper respiratory tract)

Antibiotic therapy plays no role in asthma exacerbations, unless there is clear evidence of a pulmonary infection (e. g., fever, purulent sputum, or radiologically documented infiltration).

#### Management in the emergency room/hospital

**Fig. 7:**
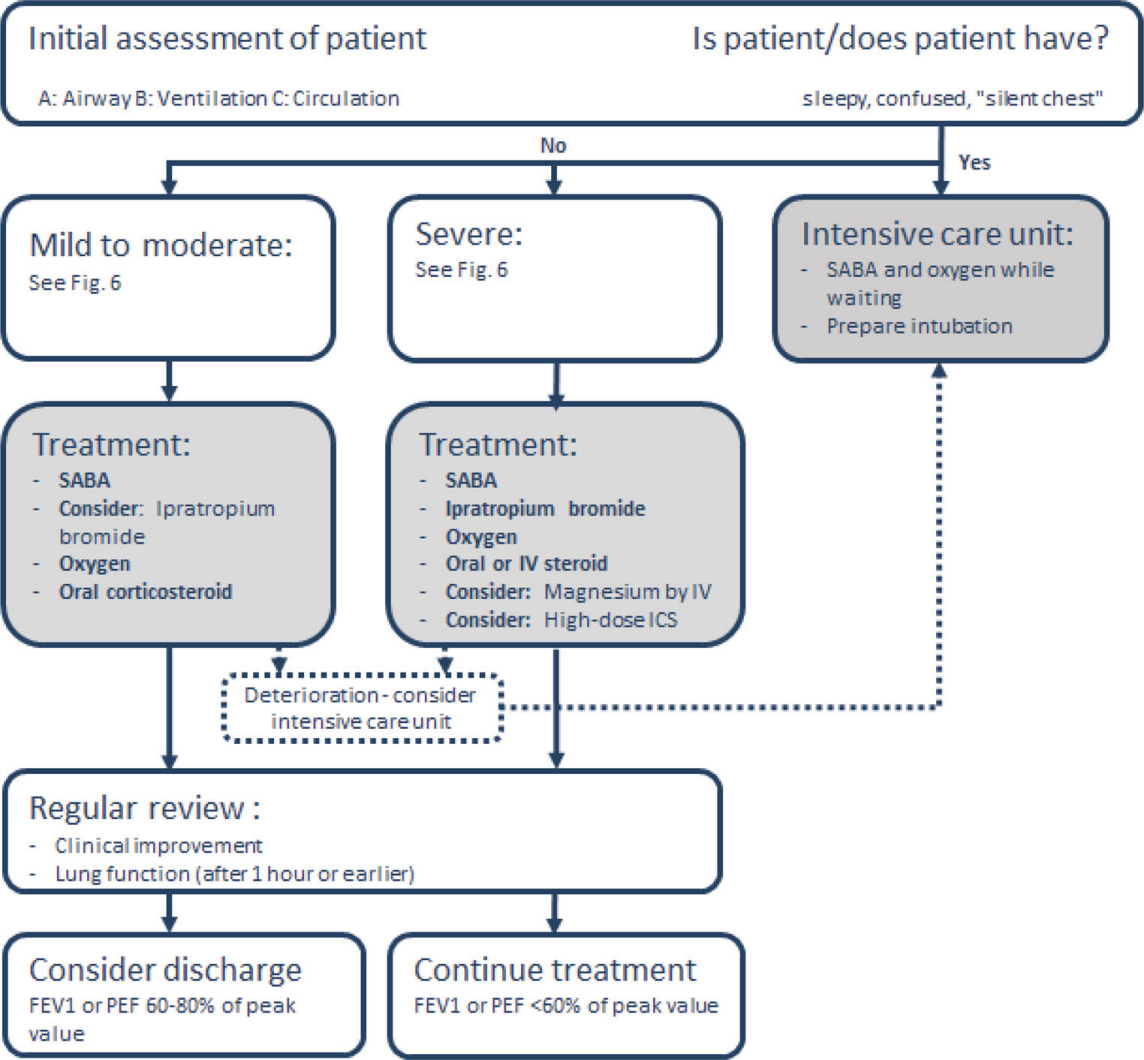
Management of asthma exacerbations in emergency rooms

Severe asthma exacerbations are life-threatening medical emergencies. Some 50–100 people still die of asthma in Austria every year (Austrian Statistics). It is usually safest to handle such emergencies in hospital emergency rooms (Fig. 7). This overview does not include any details on intensive-care management of asthma.

Analogous to the management provided by a general practitioner and/or pediatrician, the challenge is to quickly perform the three components (medical history and physical examination, assessment of severity, and prompt initiation of therapy). PEF and oxygen saturation measurement can be supplemented with a spirometry, arterial blood gas analysis and, if necessary, a chest X‑ray.

Therapies not recommended for acute care include intravenous methylxanthine (poor risk–benefit profile), helium oxygen therapy (exceptional cases only), LTRA, antibiotics, and sedatives (increased mortality). The use of noninvasive ventilation (NIV) is controversial, particularly for agitated patients. Under no circumstances should the patient be sedated to allow for NIV.

The postdischarge therapy follows the same guidelines as for management by a general practitioner or pediatrician. Patients should be promptly and repeatedly evaluated by the physician providing treatment in the weeks following the exacerbation.

### Primary prevention

Asthma is a heterogeneous disease and ultimately the result of a gene–environment interaction. According to general consensus, there is a **“**window of opportunity” during pregnancy and early childhood, in which environmental factors can modify the development of asthma. GINA briefly addresses the most comprehensively studied factors, including breastfeeding, vitamin D, supplementary food, probiotics, inhaled allergens, tobacco smoke, microbial effects, drugs (antibiotics, paracetamol), as well as psychological aspects. At the end of the chapter, GINA provides a recommendation for generally applicable advice to parents regarding *primary asthma prevention*:

- Avoid exposure to second-hand smoke, especially during pregnancy and in the child’s first year of life.
- Seek vaginal birth if possible.
- Breastfeed (generally positive health effects).
- Only use broad-spectrum antibiotics during pregnancy and in the child’s first year of life if absolutely necessary.

For the other aforementioned factors, there are either insufficient data (or partly controversial) to give a general recommendation or the approaches are not generally applicable to everyone (e. g., growing up on a farm).

## Preschoolers

For the age group 0–5 years, there are significant differences in the entire asthma management from diagnosis to therapy [2]. Therefore this group is discussed in a separate section of the GINA Guidelines.

### Diagnosis

The possibility of a first manifestation in infants or toddlers makes diagnostic determination difficult in this age group because congenital disorders and a wide range of differential diagnostic possibilities (see Table 1) have to be taken into account and may need to be investigated along with perinatal aspects.

A special feature in this age group is that many children suffer from asthma-like symptoms, while the number of children who are completely symptom-free in later life is very high. Children under 3 years of age commonly exhibit wheezing within the context of viral infections, and every third child suffers from episodic symptoms, frequently with excellent long-term prognosis. There are no reliable tests to distinguish between transient symptoms and true early childhood asthma – with the associated need for therapy.

The most significant indicators of early childhood asthma include:

- Typical symptoms such as wheezing and coughing without an infection, also frequently during exertion/laughing/crying as well as reduced activity level and particularly nocturnal symptoms
- Risk factors for asthma (especially positive family history of allergies or asthma)
- Response to a therapy attempt with low-dose ICS and SABA on demand for 2–3 months

The following additional examinations may be considered:

- *Allergy test:* Reasonable because many children show relevant sensitization even under 3 years of age. For example, sensitivity to house-dust mites is associated with increased asthma risk.
- *Lung function diagnostics*: Children usually have to be 5–6 years old before spirometry is possible, but it can be attempted earlier with sufficient expertise and time investment. The GINA Guidelines do not make any reference to alternative technologies (forced oscillation, impulse oscillometry, multiple-breath washout etc.). In addition, these techniques currently are only available in specialized centers.
- *Exhaled NO:* Listed as a possible examination (tidal technique) in the GINA Guidelines, but not yet established for young children (apart from scientific applications). As soon as spirometry is possible, the forced expiratory flow maneuver can usually be employed in a defined flow range (“single-breath” method).
- *Further tests*, mainly to rule out differential diagnoses: In addition to the lung X‑ray specified in the GINA Guidelines, further examinations (bronchoscopy, sweat test, pH measurement etc.) may be required. However, they are not part of the primary diagnosis.

### Therapy and management

Asthma control plays an important role, as in older children and adults. The GINA Guidelines offer a table on asthma control, which is similar to the table for older children and adults (see Table 2). It distinguishes between well-controlled, partly controlled, and uncontrolled asthma. In the case of young children, criteria are a little stricter (e. g., daily symptoms or SABA demand more than once a week compared with twice a week for older children; nocturnal cough is listed in addition to nocturnal awakening).

### Pharmacological therapy

**Fig. 8:**
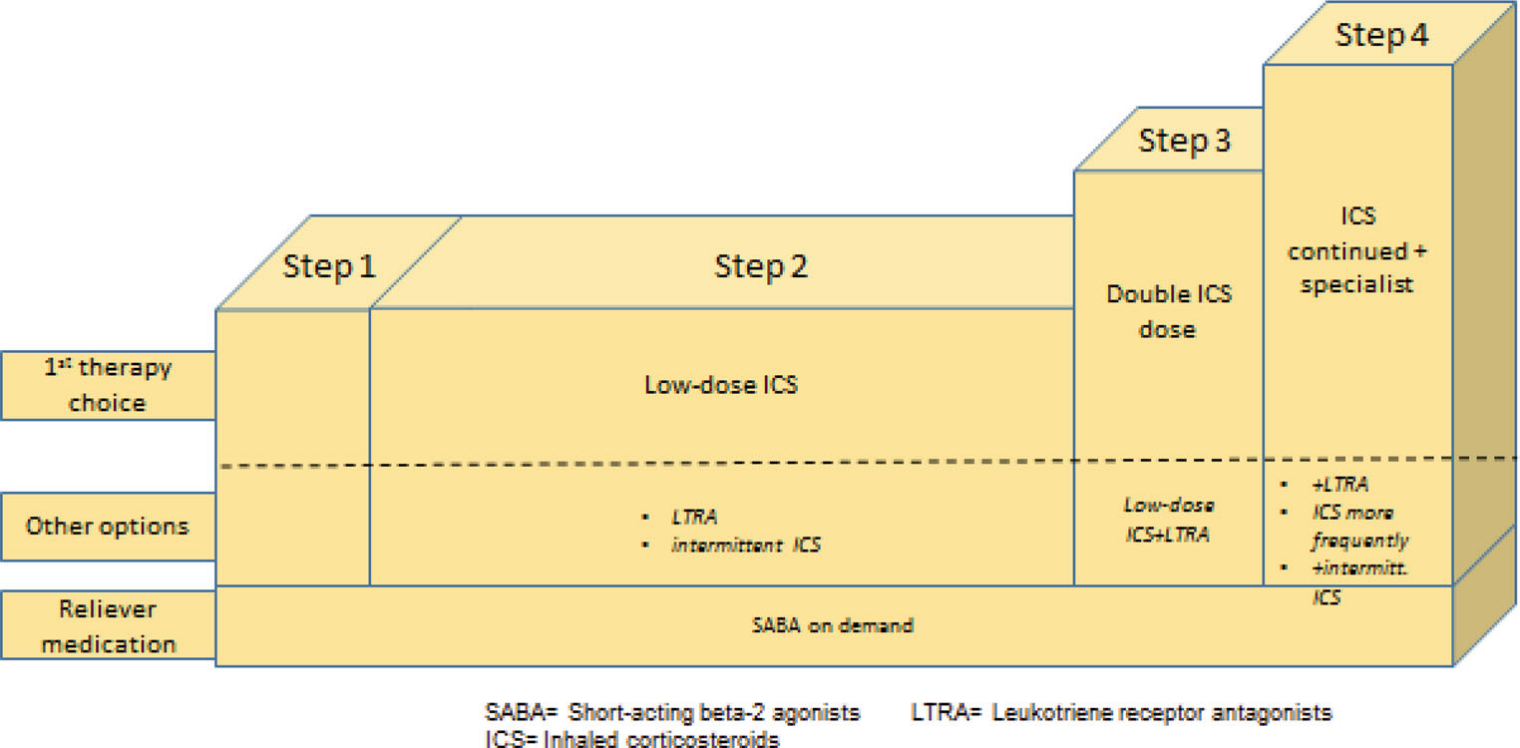
Step algorithm of asthma therapy for children ≤5 years

Pharmacological therapy follows a step algorithm, similar to older children, which is adapted to the age group (Fig. 8). An inhaled SABA on demand is the first choice in step 1; this on-demand therapy is recommended for all further therapy steps.

It is further recommended to refer the patient to a specialist from step 4 (if increasing the daily ICS dose does not lead to any improvement). Compared with previous guidelines, the administration of LTRA has declined in significance and is only recommended as an option. Current Austrian recommendations [7] also give preference to a primary therapy with low-dose ICS over an initial therapy with LTRA, since most of the children (especially those with allergies) benefit more from therapy with ICS. The GINA Guidelines also point out that oral administration of a beta-2-agonist is not recommended owing to the slower onset and higher rate of side effects.

The selection of the optimal inhaler for application of ICS and SABA is equally relevant for preschoolers because they can only properly inhale when breathing normally. The choice of inhaler is based on the child’s age and ability. The preferred device for preschoolers is a metered-dose inhaler with a spacer and valve. It should have a mask for children under 4 years of age and a mouthpiece for most children from 4 years on. It is important to know that the administered medication dose may vary considerably among different spacers. Nebulizers can be used as an alternative for the few preschool children who cannot inhale effectively in spite of practicing with a metered dose inhaler with spacer.

Regular re-evaluation and adjustment of the therapy is particularly important in this age group. Follow-up should occur within 3 months after every therapy modification.

### Asthma training and asthma plan

Asthma training also makes sense for this age group. Emphasis should be on basic information about asthma, communicating the right inhalation technique and presenting a written asthma action plan (see also “Asthma training and self-management”).

The onset of symptoms, severity, presence of differential diagnostic information, considerations concerning the inhalation technique, and, in many cases, inadequate therapeutic success frequently lead to the involvement of pediatric experts. This is highly relevant, since development-related aspects must be considered along with the fact that the absence of symptoms must be the objective of the treatment for this age group as well.

## Conclusion

Guidelines contribute to a standardized, uniform approach for the diagnosis, treatment, and management of major diseases. The GINA Guidelines have supported this effort in the area of asthma management for many years. New additions to the current guidelines include a differentiated definition of asthma that makes specific reference to the heterogeneous symptoms of the disease and no longer emphasizes bronchial hyperresponsiveness. The guidelines strongly encourage regular adaptation of the therapy regimen and the consistent reassessment of patients with different methods (clinical, lung function etc.). The role of ICS is highlighted in therapeutic aspects. These pharmaceuticals should be used at an early point in the course of the disease, preferably in low doses. ICS are now given priority over LTRA in the treatment of young children. At the highest treatment step, the concept of add-on therapy is recommended over an OCS therapy for adults. The guidelines again place particular emphasis on selecting the right inhaler and providing adequate asthma training. The implementation of the existing guidelines in routine treatment of asthma patients will be important for the future. Sufficient resources are necessary to enable training for private practitioners in hospitals as well as a comprehensive training program for asthma patients.
